# Identification of two novel hepatitis C virus subtype 2 from Tunisia (2v and 2w)

**DOI:** 10.1371/journal.pone.0248249

**Published:** 2021-03-11

**Authors:** Mouna Rajhi, Sondes Haddad-Boubaker, Anissa Chouikha, Daniel Bourquain, Janine Michel, Walid Hammami, Amel Sadraoui, Hinda Touzi, Kais Ghedira, Henda Triki

**Affiliations:** 1 Laboratory of Clinical Virology, WHO Regional Reference Laboratory for Poliomyelitis and Measles, for EMR, Pasteur Institute, Tunis, Tunisia; 2 University of Tunis El Manar, Tunis, Tunisia; 3 Research Laboratory of Virus, Vector and Host (LR20IPT10), Pasteur Institute, Tunis, Tunisia; 4 Robert Koch Institute, Centre for Biological Threats and Special Pathogens–Highly Pathogenic Viruses, Berlin, Germany; 5 Laboratory of Bioinformatics, Biomathematics and Biostatistics (LR16IPT09), Pasteur Institute, Tunis, Tunisia; Centre de Recherche en Cancerologie de Lyon, FRANCE

## Abstract

**Background:**

Hepatitis C virus (HCV) has a high genetic diversity. Eight genotypes and 90 subtypes are currently described. Genotypes are clinically significant for therapeutic management and their determination is necessary for epidemiological studies.

**Methods:**

Tunisian patients plasma samples (n = 6) with unassigned HCV-2 subtype using partial sequencing in the NS5B and Core/E1 regions were analyzed by realizing whole-genome sequencing analysis. Phylogenetic analyses were performed to assign subtypes.

**Results:**

Phylogenetic analysis of the full genome sequences of Tunisian strains shows two subtypes within HCV-2. These later were genetically distinct from all previously established HCV-2 subtypes with nucleotide divergence greater than 15% (20% -31%). These two subtypes are proposed as new subtypes 2v and 2w.

**Conclusions:**

The discovery of two new HCV-2 subtypes circulating in the Tunisian population confirms the great diversity of HCV-2 viruses and increases the total number of HCV-2 subtypes from 21 to 23.

## Introduction

Hepatitis C virus (HCV) infection constitutes one of the major public health problems. Indeed, it represents the major etiology of chronic liver diseases; cirrhosis, chronic hepatitis and hepatocellular carcinoma [1].

HCV belongs to the family of *Flaviviridae* and the genus *Hepacivirus*. Its genome consists of a single strand of positive-sense RNA that encodes a single long polyprotein [2]. HCV-2 has a high genetic diversity. Currently, eight genotypes numbered from 1 to 8 are identified and 90 subtypes are represented by lowercase letters. Genotypes differ from each other by more than 30% at the nucleotide level while subtypes are different at 15% to 25% [3, 4]. Twenty-one subtypes are currently assigned in the HCV sequence database. Fifteen subtypes (2a, 2b, 2c, 2d, 2e, 2f, 2i, 2j, 2k, 2l, 2m, 2q, 2r, 2t and 2u) are confirmed and recognized by complete genome sequencing and only six subtypes (2g, 2h, 2n, 2o, 2p, 2s) are proposed according to partial sequencing results in the NS5B and core/E1 region [4, 5]. Determination of circulating HCV genotypes is very crucial for the understanding of the epidemiology and evolution of HCV and is also clinically important for therapeutic management of patients. Even after the development of direct-acting antiviral regimens, the current American Association for the Study of Liver disease (AASLD) guidelines rely primarily on genotype [6].

Advances in sequencing technology have accelerated the rate at which HCV genome sequences are generated. Indeed, the number of confirmed genotypes has increased from 18 to 90 in 2005 and 2019 respectively. A recent study characterized 19 new HCV subtypes. However these novel sequences are not yet available in the GenBank database [3–5, 7, 8].

HCV genotypes have a worldwide distribution. HCV genotype 2 (HCV-2) is very frequent in many regions of the world such as West Africa [9], Martinique and Venezuela [10, 11]. Tunisia is a low endemic country with a prevalence of HCV infection ranging from 0.4% to 0.7%. Genotype 1 is the most common, especially subtype 1b, with prevalence of around 80% followed by genotype 2 with prevalence of 10% approximatively [12–14]. HCV genotype 2 has been poorly studied in Tunisia and until now only two studies have investigated its genetic diversity and molecular epidemiology [15, 16]. The study conducted in 2014 showed that the major circulating subtype is subtype 2c (75.5%). Some of the investigated strains were identified as belonging to HCV-2 genotype using partial sequencing of the NS5B and Core/E1 regions, while subtypes could not be identified. Thus, circulation of two new probable HCV-2 subtypes in Tunisia was suggested [15]. In the present work, we investigated Tunisian strains with unassigned HCV-2 subtype using whole genome sequence analysis.

## Materials and methods

### Ethics statement

This is a retrospective study that includes archived samples and all data were fully anonymized before we had access. Indeed, used samples were previously detected as HCV RNA-positive as part of the routine diagnostic activity for determination of viral genotype of hepatitis C virus. All patient information was anonymized and de-identified and sera were labeled with laboratory codes and finally archived. Thus, when archived samples are used, sera are identified only by laboratory codes.

### Samples

The samples included in the present study were collected from 2004 to 2008. These are sera from six Tunisian patients: four women and two men aged between 52 and 60 years with a mean age of 55 years and originated from different districts in North and Central-East of Tunisia. The samples were previously investigated using partial sequencing in the NS5B and Core/E1 regions and they belonged to HCV-2 with unassigned subtype [15].

### Sequencing

Hepatitis C virus RNA was extracted from 140 μL of serum using the QIAamp Viral RNA Mini Kit (QIAGEN, Hilden-Germany) and full HCV genome sequencing was performed at Robert Koch Institute, Centre for Biological Threats and Special Pathogens, Highly Pathogenic Viruses, Berlin, Germany. Sequences were obtained using Illumina. *De novo* assembly of HCV was performed to obtain a full genome sequence. Briefly, the sequencing data were subjected to quality control; sequence quality assessment, read filtering and mapping to a HCV reference sequence. Trimmomatic was used for pre-processing NGS reads and performs several quality filtering steps, including sequencing adapter removal, low quality base removal and discarding short reads [17]. Mapping and assembling was assessed using Bowtie2, spades and Geneious (version R9.1.3) [18–20].

### Genomes annotation

Assembled contigs were used as inputs for the genome detective web server allowing genome annotation [21, 22]. For each viral genome, nucleotide and amino acid sequences of all genes were identified. These later were integrated into HCV locator tool to identify the coordinates of gene/protein sequences in each assembled viral genome by comparison to the H77 genome sequence (AF009606) [23, 24].

### Phylogenetic analysis

Tunisian viral full-genome sequences were aligned with the reference sequences of the previously established HCV genotypes and subtypes (S1 Table). All sequences are available on the International Committee on taxonomy of viruses (ICTV) website [5].

Phylogenetic analyses were performed using maximum likelihood methods included in the MEGA tool (version 5.10). The reliability of the phylogenetic constructions was estimated by bootstrap analysis with 1,000 pseudo replicate data sets. Genetic distances were conducted using the maximum composite likelihood methods. The new full-length sequences reported in this study were submitted to GenBank under the accession numbers [GenBank: MW041294 to MW041299].

### Recombination analysis

Tunisian full-genome sequences were analyzed by Bootscan in SimPlot v.3.5.1 [25] to identify possible recombination breakpoints. Default settings were used (Step: 20 bp; GapString: On, Reps: 100, Kimura [2-parameter]; T/t: 2.0, Neighbor-Joining) and analysis was performed at the nucleotide level of genotype 1 to genotype 8 with Tunisian sequences as queries.

## Results

**Fig 1:**
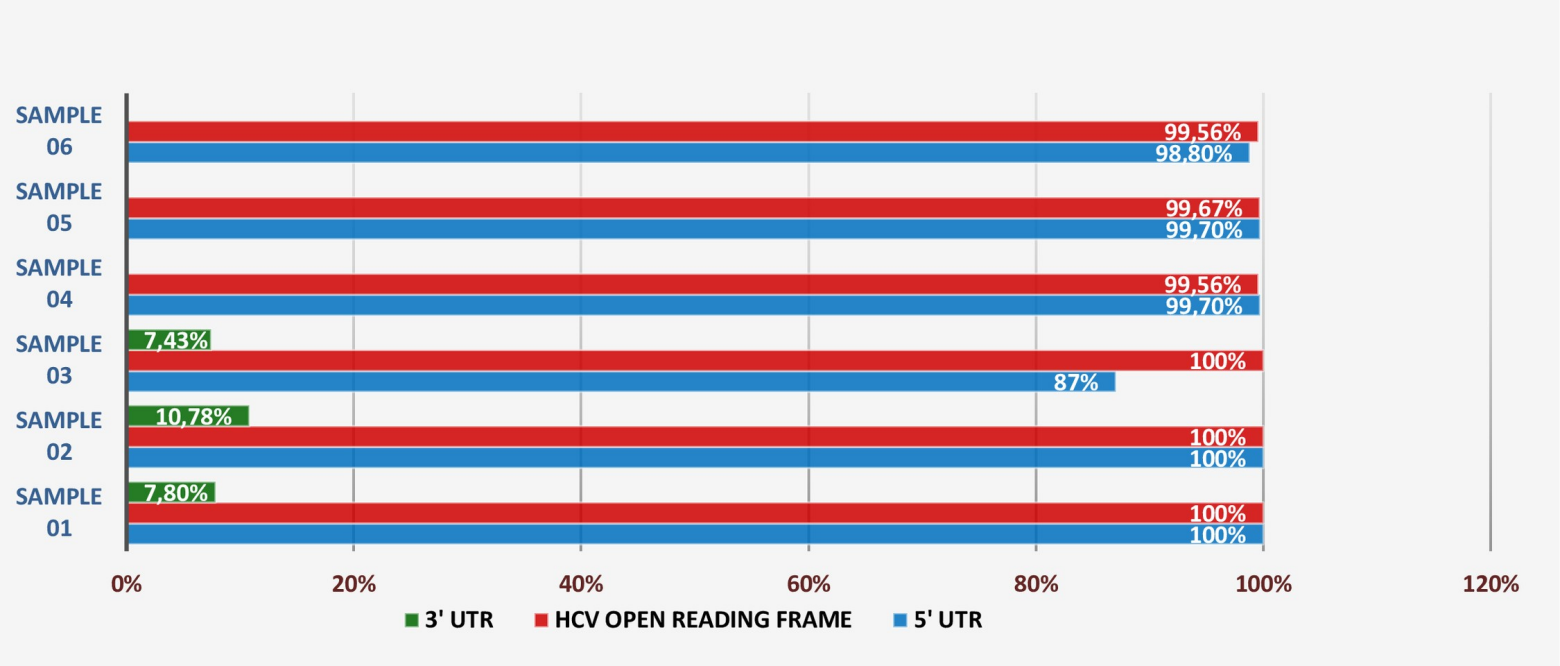
HCV regions obtained (%) by HCV full genome sequencing (compared to AF009606).

HCV genomes were successfully obtained for the six Tunisian samples investigated in the present study (Fig 1). The complete HCV sequence of the coding region was obtained for three samples (100% of total length of the coding region) and the other three sequences have truncated portions at the end of NS5B regions (99.56% - 99.67% of total length of the coding region).

The full HCV genome sequencing generated complete 5’UTR sequence for 2 samples, the other 4 sequences have truncated portions at the 5’UTR beginning (87%-99.7% of total length of 5’UTR region). Limited sequence of the 3’UTR was generated for all sequences (7.43%-10.78% of total length of 3’UTR region) (Fig 1).

For all Tunisian sequences, the lengths of E2 and NS5A regions are greater than their lengths in the reference sequences HCV-1a (AF009606). The length of E2 region of all the Tunisian sequences is about 1101nt while it is about 1089nt in HCV-1a (AF009606) reference sequence. The NS5A region has a length of 1398nt and 1401nt for the Tunisian sequences of cluster 1 and cluster 2 respectively while its length in the HCV-1a (AF009606) reference sequence is about 1344nt (S2 Table).

All assembled Tunisian sequences were aligned to HCV reference sequences (genotype 1–8). Sequences present differences in total length, therefore the 5’UTR and the NS5B regions were truncated and 3’UTR regions were removed. The final alignment covered 95.8% (9243nt /9646nt) of the total length according to the reference sequence AF009606.

**Fig 2:**
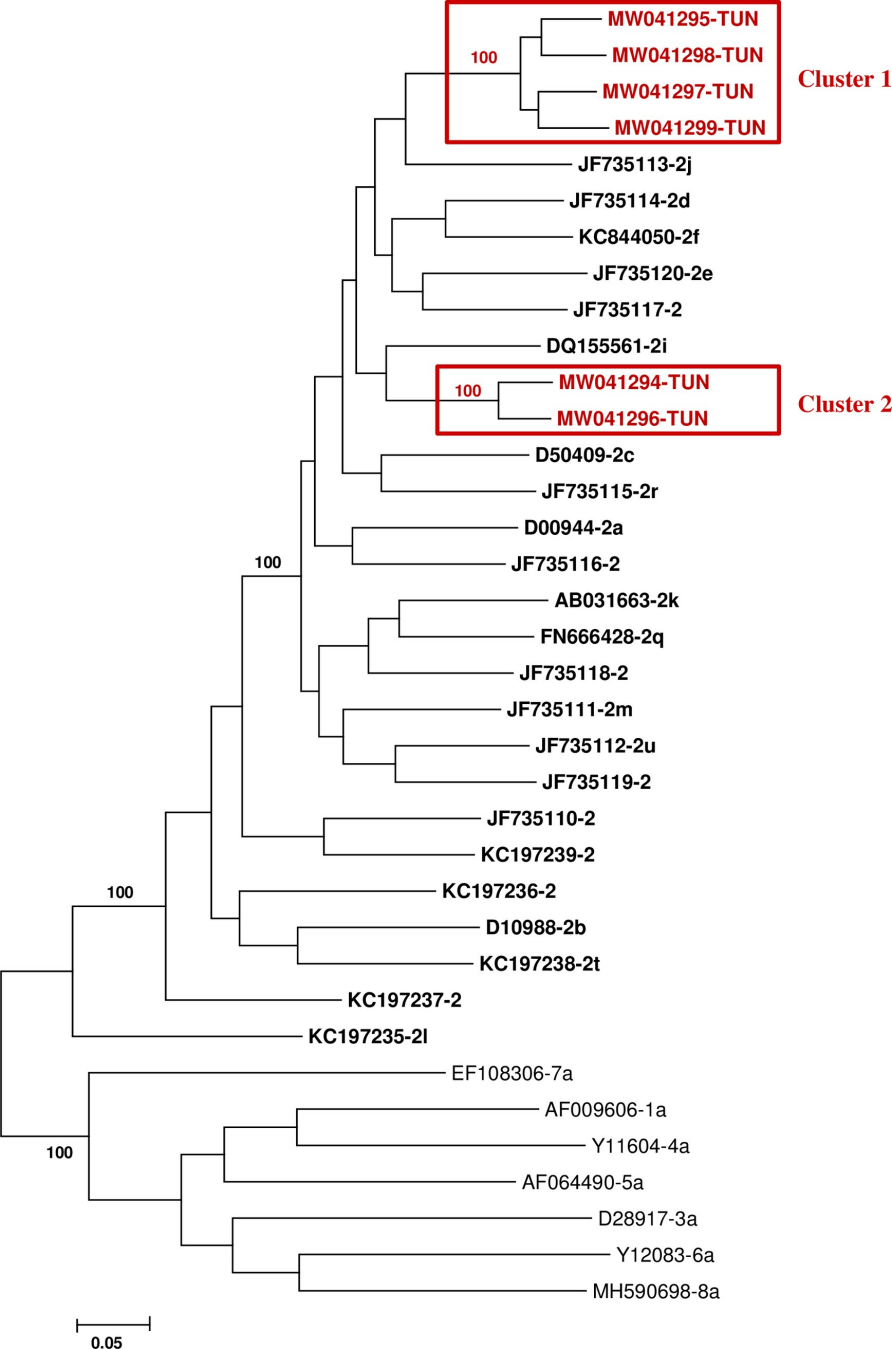
Phylogenetic analysis of Tunisian HCV-2 full genome sequences compared to previously established HCV genotypes and subtypes. The maximum likelihood phylogenetic tree was constructed with the nearly complete genome sequences (95.8% of full HCV genome, 9243nt) of the six Tunisian HCV-2 strains, the previously confirmed genotypes, confirmed HCV-2 subtypes and unassigned HCV-2 subtypes. Tunisian HCV-2 strains are highlighted in red and indicated by their GenBank accession numbers followed by the country code (TUN). The HCV genotypes sequences are indicated by their GenBank accession number followed by the corresponding letter of subtypes (unassigned HCV-2 subtypes are included without letter code) and HCV-2 references sequences are highlighted in bold. Bootstrap values were indicated in bold at tree nodes.

Phylogenetic analysis for the full genome aligned sequences was performed (Fig 2). Tunisian isolates previously typed as genotype 2 using partial sequencing in the NS5B and Core/E1 regions and with unassigned subtype were phylogenetically different from established HCV genotype 1,3,4,5,6,7 and 8.

The nucleotide divergence rates between these sequences largely exceeded 30% (41% to 47%), which is the level of divergence between currently classified HCV genotype according to the classification criteria proposed by Simmonds et al (2005) (Table 1).

**Table 1:** Nucleotide divergence rates of Cluster 1 and Cluster 2 with HCV genotype and subtype sequences.

	Divergence rates (range %)	
	Cluster 1	Cluster 2
**With HCV genotype (1,3,4,5,6,7 and 8)**	42% - 47%	41% - 46%
**With HCV-2 subtypes reference sequences**	20% - 31%	20% - 31%
**With HCV-2 unassigned subtypes**	20%-27%	20%-26%
**Cluster 1**	9% - 11%	20%-21%
**Cluster 2**	20%-21%	7%

Tunisian sequences are grouped with HCV-2 reference sequences which confirm their belonging to genotype 2. Phylogenetic analysis also shows that Tunisian strains form two phylogenetic groups within HCV-2 sequences; Cluster1 and Cluster2 that did not cluster with any of HCV-2 confirmed or unassigned subtypes previously described (Fig 2). The two clusters were supported by 100% bootstrap values and were clearly distinct from described HCV-2 subtypes. The divergence rates between Tunisian sequences are widely superior to 15% (20% - 31%) which corresponds to the level of divergence between HCV subtypes according to the classification criteria (Table 1) [3].

**Fig 3:**
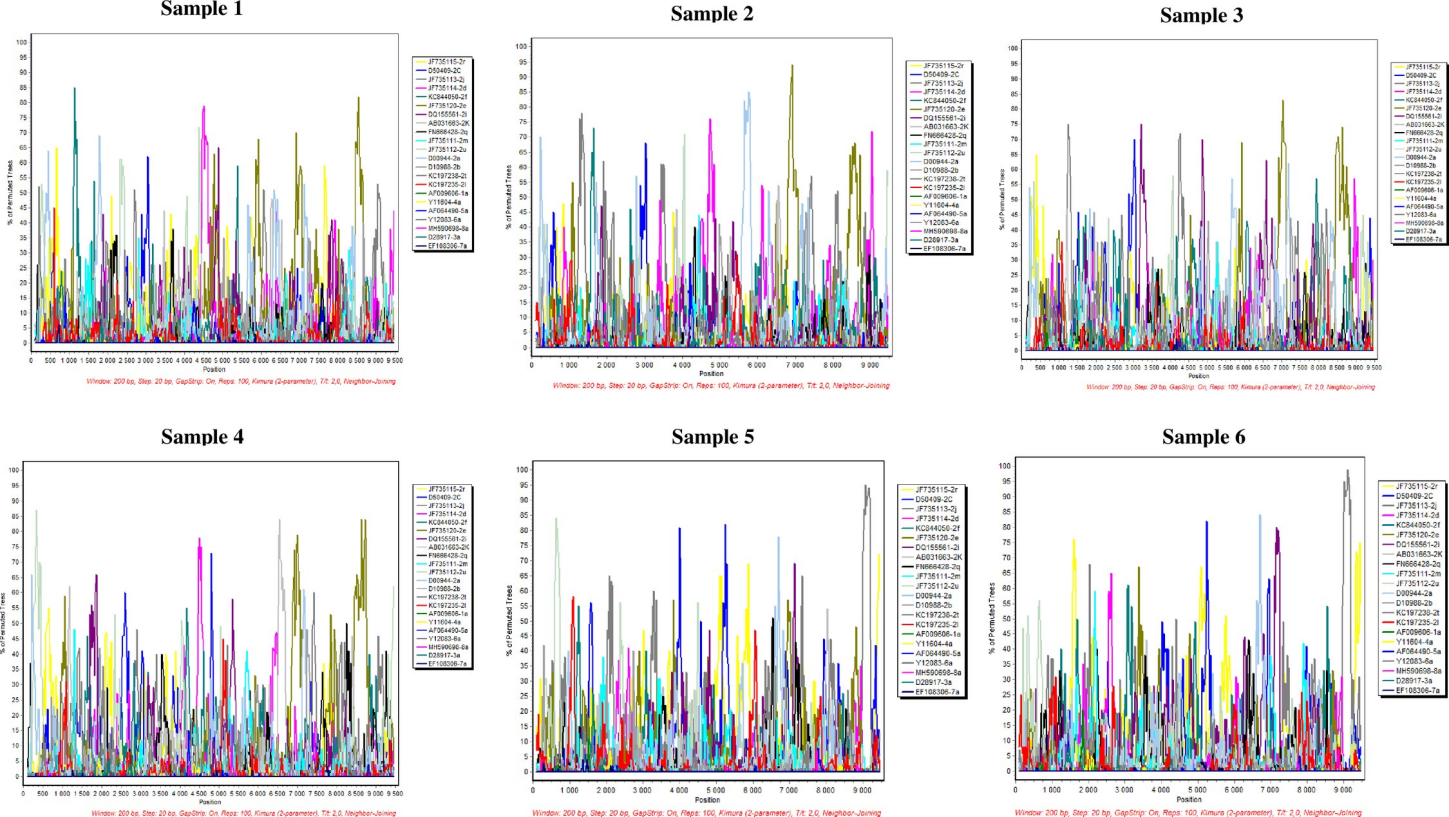
Absence of recombination in the HCV-2 Tunisian strains by BootScan analysis. BootScan analysis of nucleotide sequences of HCV-2 Tunisian strains as queries compared to previously established HCV genotypes and HCV-2 subtypes. The nearly complete genome sequences were included in the analysis (95.8% (9243nt) of full HCV genome by reference to the AF009606 sequence). Nucleotide sequence positions are indicated on the x-axis.

Cluster 1 included 4 sequences with 9%-11% nucleotide divergence between each others. Cluster 2 included 2 sequences with 7% divergence between each others (Table 1). All sequences were from patients originating from different districts without any epidemiological links. On the other hand, the assessment of Tunisian sequences by Bootscan analyses shows the absence of recombination break-point confirming that they do not represent recombinant strains with other HCV genotypes or subtypes (Fig 3). These findings suggest the classification of these Tunisian strains as two new subtypes of HCV-2 not yet described in the literature and public databases.

## Discussion

This study reports, for the first time, full genome sequence of two new HCV-2 subtypes, previously identified by partial sequencing as belonging to genotype 2 [15].

Prevalence and genetic diversity of HCV-2 varies from a country to another. It is very common in West Africa (Guinea-Bissau, Benin, Burkina Faso, and Ghana) with prevalence higher than 50% [9]. In Ghana, for example, genotype 2 is largely predominant accounting for 87% of the circulating strains and it is highly variable with the circulation of many subtypes, suggesting the endemic and ancient presence of HCV-2 within populations [26–29]. However, in Central Africa and particularly in Cameroon, genotype 2 represents only 25% of circulation strains. They are characterized by their homogeneity which could indicate a recent introduction from West Africa [30].

The great diversity and the endemic and ancient presence of HCV-2 have also been confirmed outside the African continent, in particular in Martinique and Venezuela. In these countries, genotype 2 is the second most common genotype after genotype 1 with 6.8% and 33% of prevalence respectively [10, 11].

However, in Europe, the genetic diversity and the epidemiological characteristics of the HCV-2 strains are poorly studied. In France, genotype 2 is more frequent in the southwest region with a prevalence of 11.3%, the subtypes 2i, 2k, 2c and 2a accounting for 24.7%, 22.4%, 17.4% and 10.8% respectively [31]. In Italy, genotype 2 is the second most prevalent genotype after 1b and is responsible for around 30% of cases of HCV infection; subtype 2c has the highest prevalence compared to other regions of Europe ranging from 26 to 27% in northern Italy to 45% in the south of the country [32, 33]. In North Africa, few studies have been conducted on HCV-2. Two studies carried out in Algeria and Morocco have shown that genotype 2 is the second most prevalent genotype after genotype 1 with a prevalence of 8.5% and 23% in Algeria and Morocco respectively [34, 35].

Following the development of phylogeography and molecular dating several studies have been conducted on genetic diversity, molecular epidemiology and evolution of HCV-2 in time and in space [29, 36, 37]. These studies suggested that genotype 2 is originated from Africa; the age of the most recent common ancestor (MRCA) in West Africa has been estimated around 400–600 years. Global spread of HCV-2 would have occurred during the 20th century [29, 36, 37]. The spread of HCV-2 within European populations following human migration has been highlighted. Some studies have proposed that genotype 2 would have been introduced into western countries from Africa during the slave trade and colonialism in the 17th and 18th centuries, then was transmitted mainly through blood transfusions and injection [29, 31, 38–40].

In Tunisia, genetic diversity and molecular epidemiology of HCV-2 has been poorly studied. A previous study showed that the major circulating subtype in Tunisia is subtype 2c (65.1%) with co-circulating isolates from subtypes 2k (11.2%), 2i (5.6%) and 2b (1.1%) [15]. We have previously demonstrated that the most recent common ancestor of subtype 2c was evaluated around 1886 (1869–1902), prior to the introduction of subtype 2k estimated in 1901 (1867–1931). The study suggests that the introduction of HCV-2c in Tunisia is possibly a result of population movements between Tunisian and European population especially between countries of the Mediterranean basin following the French colonization [16].

Tunisian patients enrolled in this study were previously identified as infected with HCV-2 without any subtypes identification. Thus, a probable circulation of two new HCV-2 subtypes was suggested [15]. In the present study, results of full genome sequencing confirmed the belonging of Tunisian strains to two new subtypes within HCV genotype 2. These results are consistent with assignments rules of a new HCV subtype proposed in the latter consensus proposal. Therefore, we propose the classification of these Tunisian isolates as subtypes 2v and 2w.

The discovery of two new HCV-2 subtypes circulating in Tunisian population confirms the great diversity within HCV-2. Therefore, the total number of subtypes will increase from 21 to 23. This finding might suggest establishment and endemic circulation of HCV-2 genotypes for a long period of time. It has also important implications for the genetic and epidemiological characterization of the HCV-2 worldwide. Genotype determination is also crucial for determining the appropriate clinical treatment.

## Conclusion

We report the finding of two new HCV-2 subtypes identified in 6 patients originating from Tunisia, which confirms the circulation of these newly identified lineages in the human population and approves the great diversity within HCV-2. The total number of subtypes will then increase from 21 to 23.

## Supporting information

S1 Table HCV reference sequences used in the phylogenetic analyses. (DOCX)

S2 Table HCV regions obtained by HCV full genome sequencing (compared to reference sequence AF009606). (DOCX)
