# Supplementary material for: Identification of two novel hepatitis C virus subtype 2 from Tunisia (2v and 2w)
Source: PLoS One. 2021 Mar 11;16(3):e0248249. doi: 10.1371/journal.pone.0248249 (PMC7951806; doi:10.1371/journal.pone.0248249)
Supplement: S1 Table — (DOCX) [file pone.0248249.s001.docx]

| **Accession numbers** | **Genotypes** | **References** |
| --- | --- | --- |
| AF009606 | 1a | Kolykhalov AA et al 1997 |
| D00944 | 2a | Kato et al*,* 2001 |
| D10988 | 2b | Murakami et al*,* 2001 |
| D50409 | 2c | Nakao et al*,* 1996 |
| JF735114 | 2d | Li et al*,* 2012 |
| JF735120 | 2e | Li et al*,* 2012 |
| KC844050 | 2f | Xu et al*,* 2013 |
| DQ155561 | 2i | Noppornpanth et al*,* 2006 |
| JF735113 | 2j | Li et al*,* 2012 |
| AB031663 | 2k | Samokhvalov et al*,* 2000 |
| KC197235 | 2l | Jordier et al*,* 2013 |
| JF735111 | 2m | Li et al*,* 2012 |
| FN666428 | 2q | Martró et al*,* 2011 |
| JF735115 | 2r | Li et al*,* 2012 |
| KC197238 | 2t | Jordier et al*,* 2013 |
| JF735112 | 2u | Li et al*,* 2012 |
| JF735119 | 2 | Li et al*,* 2012 |
| JF735110 | 2 | Li et al*,* 2012 |
| JF735117 | 2 | Li et al*,* 2012 |
| JF735116 | 2 | Li et al*,* 2012 |
| JF735118 | 2 | Li et al*,* 2012 |
| KC197236 | 2 | Jordier et al*,* 2013 |
| KC197237 | 2 | Jordier et al*,* 2013 |
| KC197239 | 2 | Jordier et al*,* 2013 |
| D28917 | 3a | Yamada et al*,* 1994 |
| Y11604 | 4a | Chamberlain et al*,* 1997 |
| AF064490 | 5a | Chamberlain et al*,* 1997 |
| Y12083 | 6a | Tokita et al*,* 1998 |
| EF108306 | 7a | Murphy et al*,* 2015 |
| MH590698 | 8a | Borgia et al*,* 2018 |
