# Supplementary material for: Identification of two novel hepatitis C virus subtype 2 from Tunisia (2v and 2w)
Source: PLoS One. 2021 Mar 11;16(3):e0248249. doi: 10.1371/journal.pone.0248249 (PMC7951806; doi:10.1371/journal.pone.0248249)
Supplement: S2 Table — (DOCX) [file pone.0248249.s002.docx]

|  | **Sample 1** | **Sample 2** | **Sample 3** | **Sample 4** | **Sample 5** | **Sample 6** | **HCV reference sequence AF009606** |
| --- | --- | --- | --- | --- | --- | --- | --- |
| **5’UTR** | 341nt | 341nt | 297nt | 340nt | 340nt | 337nt | **341nt** |
| **Core** | 573nt | 573nt | 573nt | 573nt | 573nt | 573nt | **573nt** |
| **E1** | 576nt | 576nt | 576nt | 576nt | 576nt | 576nt | **576nt** |
| **E2** | 1101nt | 1101nt | 1101nt | 1101nt | 1101nt | 1101nt | **1089nt** |
| **P7** | 189nt | 189nt | 189nt | 189nt | 189nt | 189nt | **189nt** |
| **NS2** | 651nt | 651nt | 651nt | 651nt | 651nt | 651nt | **651nt** |
| **NS3** | 1893nt | 1893nt | 1893nt | 1893nt | 1893nt | 1893nt | **1893nt** |
| **NS4A** | 162nt | 162nt | 162nt | 162nt | 162nt | 162nt | **162nt** |
| **NS4B** | 783nt | 783nt | 783nt | 783nt | 783nt | 783nt | **783nt** |
| **NS5A** | 1398nt | 1398nt | 1398nt | 1398nt | 1401nt | 1401nt | **1344nt** |
| **NS5B** | 1776nt | 1776nt | 1776nt | 1737nt | 1747nt | 1737nt | **1776nt** |
| **3’UTR** | 21nt | 29nt | 20nt | 0nt | 0nt | 0nt | **269nt** |
